# Nanoluciferase Reporter Gene System Directed by Tandemly Repeated Pseudo-Palindromic NFAT-Response Elements Facilitates Analysis of Biological Endpoint Effects of Cellular Ca^2+^ Mobilization

**DOI:** 10.3390/ijms19020605

**Published:** 2018-02-18

**Authors:** Wei Zhang, Terunao Takahara, Takuya Achiha, Hideki Shibata, Masatoshi Maki

**Affiliations:** Department of Applied Molecular Biosciences, Graduate School of Bioagricultural Sciences, Nagoya University, Furo-cho, Chikusa-ku, Nagoya 464-8601, Japan; zhang.wei@i.mbox.nagoya-u.ac.jp (W.Z.); achiha.takuya@b.mbox.nagoya-u.ac.jp (T.A.); shibabou@agr.nagoya-u.ac.jp (H.S.)

**Keywords:** ALG-2, calcium-binding protein, calcineurin, calcium signaling, carbachol, nanoluciferase, NFAT, PDCD6, reporter gene assay, SOCE, transcription factor

## Abstract

NFAT is a cytoplasm-localized hyper-phosphorylated transcription factor that is activated through dephosphorylation by calcineurin, a Ca^2+^/calmodulin-dependent phosphatase. A non-palindromic NFAT-response element (RE) found in the *IL2* promoter region has been commonly used for a Ca^2+^-response reporter gene system, but requirement of concomitant activation of AP-1 (Fos/Jun) often complicates the interpretation of obtained results. A new nanoluciferase (NanoLuc) reporter gene containing nine-tandem repeats of a pseudo-palindromic NFAT-RE located upstream of the *IL8* promoter was designed to monitor Ca^2+^-induced transactivation activity of NFAT in human embryonic kidney (HEK) 293 cells by measuring luciferase activities of NanoLuc and co-expressed firefly luciferase for normalization. Ionomycin treatment enhanced the relative luciferase activity (RLA), which was suppressed by calcineurin inhibitors. HEK293 cells that stably express human STIM1 and Orai1, components of the store-operated calcium entry (SOCE) machinery, gave a much higher RLA by stimulation with thapsigargin, an inhibitor of sarcoplasmic/endoplamic reticulum Ca^2+^-ATPase (SERCA). HEK293 cells deficient in a penta-EF-hand Ca^2+^-binding protein ALG-2 showed a higher RLA value than the parental cells by stimulation with an acetylcholine receptor agonist carbachol. The novel reporter gene system is found to be useful for applications to cell signaling research to monitor biological endpoint effects of cellular Ca^2+^ mobilization.

## 1. Introduction

NFAT, a name after “a nuclear factor of activated T cells”, is a transcription factor containing a REL-homology region (RHR). NFAT is expressed not only in lymphocytes but also in many different types of cells and tissues, and is involved in immune system regulation, development, cancer progression and apoptosis [1,2,3,4,5]. Among the five NFAT genes (*NFAT1-5*) in mammals, four types of NFATs (*NFAT1-4*) are hyper-phosphorylated and retained in the cytoplasm in the resting state. Subsequent to Ca^2+^ mobilization upon cell stimulation, NFAT is dephosphorylated by a Ca^2+^-calmodulin (CaM)-activated protein phosphatase, calcineurin (the reaction inhibited by FK506 and cyclosporine A) [6,7], and exposes a nuclear localization signal to translocate from the cytoplasm to the nucleus [8]. NFAT generally cooperates with other transcription factor partners to regulate transcription by binding to a so-called NFAT-response element (RE) found in certain genes [1]. The most frequently used NFAT-RE reporter system has a composite non-palindromic enhancer element derived from the *IL2* promoter. An NFAT monomer and AP-1 (Fos/Jun heterodimer) bind in a quaternary complex to this element [9]. When using the *IL2* NFAT-RE, stimulation of cells by Ca^2+^-mobilizing agents such as ionomycin (a Ca^2+^ ionophore) is not sufficient. It is necessary to activate AP-1 by protein kinase C (PKC) activators such as phorbol 12-myristate 13-acetate (PMA, also named 12-*O*-tetradecanoyl-phorbol-13-acetate, TPA) [10,11]. The PKC activation leads to induction of Fos and Jun as well as phosphorylation of Jun by Jun-N-terminal kinase (JNK) activation and enhances transcription driven by TPA response element (TRE, alias AP1-RE) [12,13]. Moreover, PKCs are known to directly regulate NFATs by phosphorylation [14,15,16] and also affect intracellular Ca^2+^ levels by phosphorylating the Ca^2+^ channel Orai1 or other factors [16,17]. Thus, concomitant use of PMA in addition to Ca^2+^-mobilizing agents in experimental conditions complicates the interpretation of obtained results and restricts the application of the *IL2* NFAT-RE reporter system to the Ca^2+^ signaling studies.

To overcome the above-mentioned practical problems, utilization of an NFAT-RE system independent of partnering transcription factors is required. NFAT1 homodimers have been shown to bind κB-like sites in HIV-1 LTR [18] and in promoters of the *IL8*, *TNFα*, and *Grail* genes [19,20,21]. The *IL8* promoter upstream region has a pseudo-palindromic (5′-GGAATTTCC-3′) NFAT-RE, which is essential for driving luciferase reporter gene expression by stimulation with thapsigargin, a sarcoplasmic reticulum/endoplasmic reticulum (SR/ER) Ca^2+^-ATPase (SERCA) inhibitor causing an elevation of cytosolic Ca^2+^ concentration in breast cancer cells [22]. Therefore, we focused on the pseudo-palindromic sequence of the *IL8* promoter, and to increase the reporter sensitivity, we used nanoluciferase (NanoLuc; Nluc) that is a 19-kDa catalytic subunit from the deep sea shrimp luciferase and has been engineered to produce glow-type luminescence capable of more efficient light emission using a novel substrate, furimazine [23]. NanoLuc has a specific activity ~150-fold greater than that of firefly luciferase (Fluc) and sea pansy *Renilla* luciferase (Rluc).

We designed a NanoLuc reporter gene containing nine tandem repeats of the *IL8* NFAT-RE in the region upstream of a minimum promoter in a commercially available vector. The Ca^2+^-dependent NanoLuc expression system was evaluated in human embryonic kidney (HEK) 293 cells by three basic criteria: dependency on Ca^2+^-mobilizing reagents, inhibition by calcineurin inhibitors, and enhancement by exogenous expression of NFATs. The NanoLuc activity by endogenous NFATs was low, but it was significantly enhanced by stably expressing human STIM1 and Orail, components of the store-operated Ca^2+^ entry (SOCE) machinery or Ca^2+^-release activated Ca^2+^ (CRAC) channels [24,25,26]. By stimulation with an acetylcholine receptor agonist, a higher NanoLuc activity by endogenous NFATs was observed in HEK293 cells deficient in ALG-2 (gene name: *PDCD6*), a penta-EF-hand Ca^2+^-binding protein that has been reported to interact with a variety of intracellular proteins associated with membrane trafficking, apoptosis and Ca^2+^ homeostasis modulation [27,28,29,30,31,32,33,34,35,36,37]. The newly developed NanoLuc reporter system is found to serve as a useful tool for Ca^2+^ signaling research.

## 2. Results

### 2.1. Construction of a New NFAT-Response Element (RE)-Directed Reporter Gene

**Figure 1:**
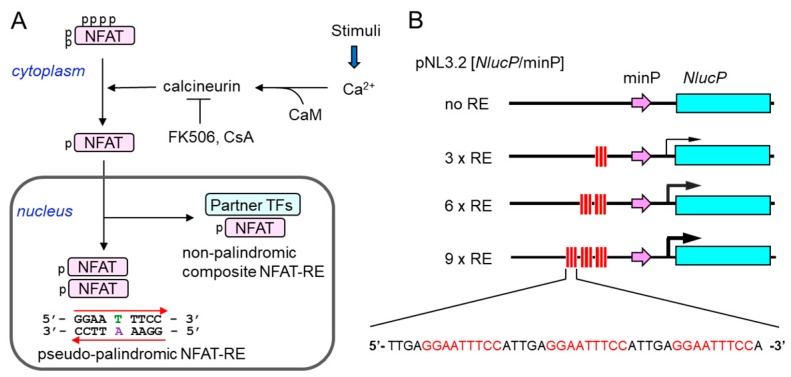
Construction of a pseudo-palindromic NFAT-response element (RE)-directed nanoluciferase (Nanoluc) reporter system. (**A**) Under the resting cell condition, NFAT is hyper-phosphorylated (indicated by p) and localized in the cytoplasm in an inactive conformation. Subsequent to cell stimulation-induced cytosolic Ca^2+^ elevation, NFAT is dephosphorylated by Ca^2+^-calmodulin (CaM)-activated protein phosphatase calcineurin and translocated to the nucleus to regulate gene expression in the immune and non-immune systems. NFAT binds either to a non-palindromic composite NFAT-RE by cooperating with partner transcription factors (TFs) or to a pseudo-palindromic NFAT-RE as a dimer. Immuno-suppressants FK506 and cyclosporine A (CsA) suppress the NFAT activation by inhibiting calcineurin. (**B**) Schematic representation of a new luciferase reporter. A pseudo-palindromic NFAT-RE found in the *IL8* gene is tandemly placed (3×, 6×, and 9×) upstream of the minimum promoter (minP) that drives transcription of the NanoLuc reporter gene *NlucP* in the basic reporter vector pNL3.2[*NlucP*/minP].

To indirectly monitor effects of increases in the intracellular Ca^2+^ concentrations, we developed a series of Ca^2+^-inducible NanoLuc reporters based on the Ca^2+^-dependent activation of dimeric NFAT (Figure 1A). The tested reporters contain different numbers of tandem repeats (none, 3×, 6×, and 9×) of a pseudo-palindromic NFAT-RE of the *IL8* promoter (5′-GGAATTTCC-3′) [19,22], which should drive transcription of the NanoLuc reporter gene *NlucP* (Figure 1B and Figure S1).

HEK293 cells were co-transfected with expression plasmids for murine NFAT1, Fluc and each newly designed NanoLuc reporter. The Fluc expression plasmid was used to normalize transfection efficiency. Cell lysates were used to measure luminescent signals of Nluc and Fluc using a Nano-Glo^®^ Dual-Luciferase Reporter Assay System provided from Promega (Fitchburg, WI, USA). The obtained raw data were normalized and presented as relative luciferase activity (RLA), which was the calculated ratio of the Nluc activity to the Fluc activity (Nluc/Fluc).

**Figure 2:**
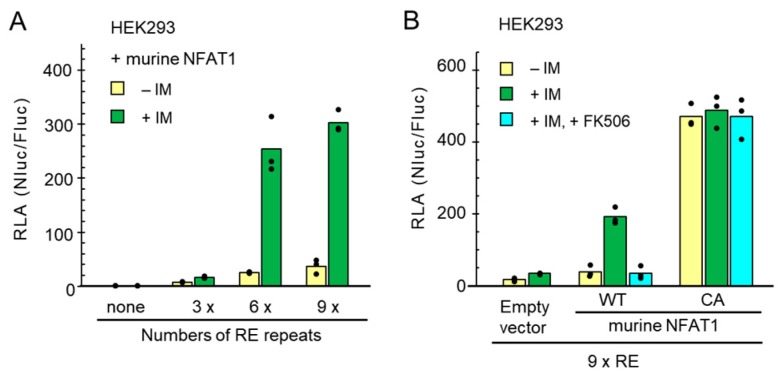
Evaluation of the NanoLuc reporter system by expressing murine NFAT1. (**A**) HEK293 cells were co-transfected with an expression vector of murine NFAT1, a firefly luciferase (Fluc) expression plasmid (pGL4.53[*luc2/*PGK]), and pNL3.2[*NlucP/*minP] containing different numbers (none, 3, 6 and 9) of the *IL8* NFAT-RE. One day after transfection, cells were stimulated with ionomycin (IM, 1 μM; vehicle, 0.007% ethanol) for 6 h. Cell lysates were used to measure luminescent signals of NanoLuc (Nluc) and Fluc using a Nano-Glo Dual-Luciferase Reporter Assay System. The ratio of Nluc to Fluc, Nluc/Fluc, is expressed as normalized relative luciferase activity (RLA). Dots and bars represent individual and averaged RLA values obtained from triplicate assays, respectively. (**B**) HEK293 cells were co-transfected with expression plasmids for NanoLuc reporter and Fluc together with either wild type (WT), constitutively active type (CA) murine NFAT1 or empty vector (pcDNA3). One day after transfection, cells were pre-treated with FK506 (10 μM) or vehicle (0.16% ethanol) for 1 h and then subjected to ionomycin (IM) stimulation for 6 h, followed by luciferase assays.

As shown in Figure 2A, under unstimulated conditions, the value of RLA increased slightly along with the numbers of RE repeats. Upon stimulation with ionomycin (IM, 1 μM) for 6 h, the RLA value increased slightly by 3×RE but significantly by 6×RE and 9×RE. Next, to verify that the NanoLuc reporter was activated through the Ca^2+^-regulated calcineurin/NFAT signaling pathway, the reporter assay using the 9×RE reporter was performed in the absence or presence of calcineurin inhibitor FK506 (Figure 2B). The ionomycin-stimulated transactivation by the exogenously expressed murine wild type (WT) NFAT1 was suppressed to the unstimulated level by pre-treatment of the cells with 10 μM FK506 for 1 h before stimulation with ionomycin. The transactivation was also similarly inhibited by pre-treatment with cyclosporine A (CsA) (Figure S2). On the other hand, the murine constitutively active (CA) NFAT1 mutant, in which 12 serine residues in the multiple regulatory phosphorylation sites in the wild type were mutated to alanines [8], exhibited a greater increase in the RLA value even without stimulation with ionomycin, and pre-treatment with FK506 did not suppress the transactivation activity. Low concentrations of ethanol used as vehicles of calcineurin inhibitors (ethanol: 0.1%, CsA; 0.16%, FK506) showed a little suppressive effect on the ionomycin-dependent reporter assay (Figure S2). Since ethanol itself did not inhibit the reporter activities under unstimulated conditions (Figure S3), ethanol may cause small adverse effects in combination with ionomycin. Although the value was very small, an increase in RLA caused by ionomycin treatment was also observed in HEK293 cells without exogenous NFAT expression (Figure 2B, Empty vector, and Figure S4A). Due to unknown reasons, however, the 6×RE reporter showed higher RLA values than the 9×RE reporter in the absence of exogenously expressed murine NFAT1 under both ionomycin-stimulated and -unstimulated conditions in HEK293 cells (Figure S4A). However, the calculated stimulation rate (fold stimulation: the ratio of RLA of the stimulated state and that of the unstimulated state) was greater using the 9×RE reporter than the 6×RE reporter (Figure S4A). These results indicate that the novel NanoLuc reporter assay system of the 9×IL8 NFAT-RE provides a potentially useful tool to monitor biological endpoint effects of the increase of cytosolic Ca^2+^ level by measuring transactivation activity of NFAT in HEK293 cells.

### 2.2. Application of the NanoLuc Reporter to Analyze Ca^2+^ Influx through CRAC Channels

SOCE is a process contributing to the elevation of intracellular Ca^2+^ concentrations by Ca^2+^ influx from the extracellular space through CRAC channels in response to the depletion of Ca^2+^ store in the endoplasmic reticulum (ER), followed by re-filling of the ER with Ca^2+^ to maintain Ca^2+^ homeostasis [24,25,26]. The functional CRAC channels are assembled from the Ca^2+^ store depletion sensor STIM1 (ER-resident stromal interaction molecule 1) and the plasma membrane Ca^2+^ channel named Orai1. NFAT has been reported as a downstream transcription factor of Ca^2+^ influx through CRAC channels [38,39]. We investigated whether the newly designed 9×IL8 NFAT-RE-directed NanoLuc expression system was suitable for detecting an intracellular Ca^2+^ increase caused by SOCE as biological endpoint effects. First, we established HEK293 cells that stably overexpress human STIM1 and Orai1 (HEK293 S1/O1) by the retrovirus expression system.

**Figure 3:**
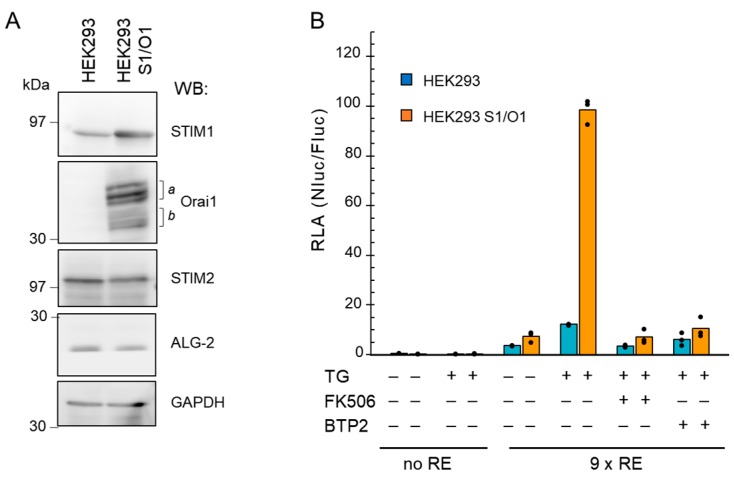
Detection of the NanoLuc reporter activity by endogenous NFAT in HEK293 cells depending on Ca^2+^-influx through CRAC channels. (**A**) HEK293 cells stably expressing human STIM1 and Orai1 (HEK293 S1/O1) were established by the retrovirus vector system as described in the Materials and Methods. Total lysates of the parental HEK293 and S1/O1 cells were analyzed by Western blotting (WB) with antibodies against STIM1, Orai1, STIM2, ALG-2, and GAPDH (used as a loading control), respectively. Multiple bands indicated by half-square brackets *a* and *b* probably reflect differences in glycosylated forms of Orai1. (**B**) One day after transfection with the 9×IL8 NFAT-RE NanoLuc reporter plasmid and Fluc plasmid, the parental HEK293 and S1/O1 cells were pre-treated with or without FK506 (10 μM) or CRAC channel inhibitor BTP2 (1 μM; vehicle, 0.1% DMSO) for 1 h, and then stimulated with thapsigargin (TG, 200 nM; vehicle, 0.02% DMSO) for 6 h, followed by luciferase assays. A NanoLuc reporter containing no NFAT-RE (no RE) was used as a negative control. Dots and bars represent individual and averaged values obtained from triplicate assays, respectively. The presence and absence of the reagents are indicated by + and −, respectively.

As confirmed by Western blot (WB) analysis using GAPDH as a loading control (Figure 3A), compared to the parental HEK293 cells, both STIM1 and Orai1 were highly overexpressed in the HEK293 S1/O1 cells. Multiple bands of Orai1 including faster migrating bands detected with anti-Orai1 (indicated by half-square brackets *a* and *b*) may account for differences in glycosylation forms since treatment of the lysate with PNGase F caused a shift from slower-migrating bands to faster-migrating bands (Figure S5). Specific bands for Orai1 were not detected by WB in the parental HEK293 cells. No significant changes in the amounts of ALG-2, a penta-EF-hand Ca^2+^-binding protein [33], were found between the two cell lines, whereas intensity of the WB signal for STIM2 was slightly reduced. Next, we performed the reporter assay under the SOCE induction condition. Treatment with thapsigargin, a well-known SERCA inhibitor, causes the ER luminal Ca^2+^ depletion and subsequent SOCE in turn by activation of CRAC channels [40]. As shown in Figure 3B, upon stimulation with thapsigargin (TG, 200 nM), a striking increase in NFAT transactivation activity expressed as RLA was observed in the HEK293 S1/O1 cells with the 9×RE reporter, but not with the control reporter (containing no RE). The increase of the RLA value was suppressed by pre-treatment of the cells with FK506 (10 μM). Moreover, the NanoLuc reporter activity was inhibited by 1 μM of BTP2 (alias, YM-58483), an analog of 3,5-*bis*(trifluoromethyl) pyrazole (BTP) that was first developed as a potent inhibitor of the *IL2* gene expression and NFAT activation [41] and was later found to inhibit CRAC channels [42,43]. Although the increase of the RLA value by thapsigargin was small in the parental HEK293 cells, FK506 and BTP2 showed similar inhibitory effects. These results indicate that the 9×IL8 NFAT-RE NanoLuc reporter can be used to monitor effects of Ca^2+^-influx through CRAC channels.

### 2.3. Enhanced NanoLuc Response by Carbachol Stimulation in ALG-2 Deficient Cells

We asked whether the 9×IL8 NFAT-RE NanoLuc reporter system can be also used to detect intracellular Ca^2+^ increase induced by physiological stimulants. HEK293 cells are known to respond to neuronal receptor agonists such as carbachol (an acetylcholine analog for acetylcholine receptor, AChR) and ATP [44,45,46]. Palty et al. [47] reported that SARAF, a negative regulator of SOCE, suppressed SOCE in HEK293T cells when cells were stimulated with ATP plus carbachol. Since SARAF was found to contain a binding motif for a Ca^2+^-binding protein ALG-2 [48], first we investigated effects of carbachol (CCh, 100 μM) plus ATP (100 μM) in HEK293 cells as well as in the previously established HEK293 ALG-2 knockdown (KD) cell line (ALG-2KD), in which expression of ALG-2 was suppressed by stable expression of short hairpin (sh) RNA targeting the ALG-2 mRNA [49].

**Figure 4:**
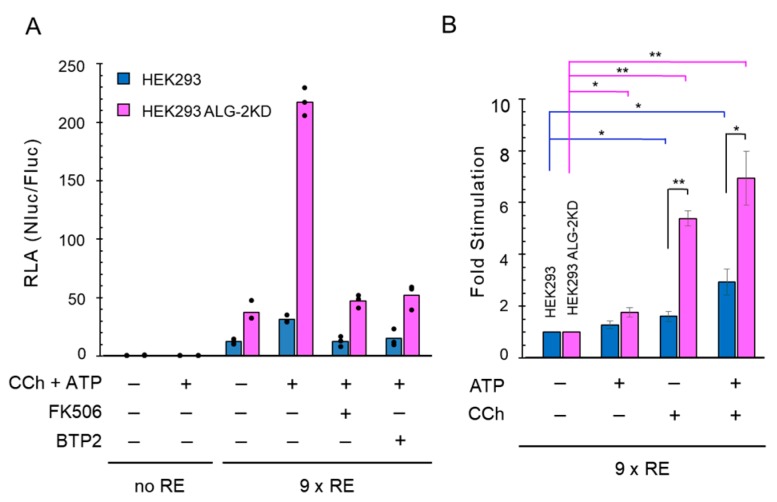
A higher NFAT activation by carbachol stimulation in the ALG-2KD cells than the parental HEK293 cells. (**A**) NFAT reporter assays were performed in triplicates using the 9×IL8 NFAT-RE NanoLuc reporter by concomitant stimulation with carbachol (CCh, 100 μM; vehicle, medium) and ATP (100 μM; vehicle, medium) for 6 h with or without pre-treatment with FK506 (10 μM) or BTP2 (1 μM) for 1 h. Dots and bars represent individual and averaged values obtained from triplicate assays, respectively. The presence and absence of the reagents are indicated by + and −, respectively. (**B**) The NFAT reporter assays in triplicates were performed as in (**A**) by stimulation with ATP (100 μM) and carbachol (CCh, 100 μM) separately or concomitantly. Independent experiments in triplicates were performed three times under the same conditions. Fold stimulation represents the ratio of RLA values obtained by stimulation to those obtained without stimulation (the resting condition) and is presented as mean ± SEM (*n* = 3). Statistical significance by Tukey’s test is indicated by asterisks (*, *p* < 0.05; **, *p* < 0.01).

As shown in Figure 4A, the RLA values using the 9×IL8 NFAT-RE reporter increased markedly compared to the reporter containing no RE (9×RE vs. no RE) in both cell lines under the unstimulated condition, and was further augmented by stimulation with carbachol plus ATP. The agonist-mediated augmentation was strongly abolished by pre-treatment of the cells with either FK506 or BTP2, respectively. Furthermore, the RLA values were higher in the ALG-2KD cells than in the parental cells both in the resting state and in the stimulated state. Next, we investigated the effects of ATP and carbachol individually and statistically analyzed the data obtained from three independently performed reporter assays. The stimulatory effect expressed as fold stimulation was much greater by treatment with carbachol alone (~5.4 fold) than by ATP alone (~1.8 fold) in the ALG-2KD cells (Figure 4B), whereas carbachol stimulated only ~1.5 fold and no significant stimulation was observed by ATP in the parental HEK293 cells.

### 2.4. No Significant Effects of ALG-2 Deficiency on AP1-RE and cAMP-RE Reporters

**Figure 5:**
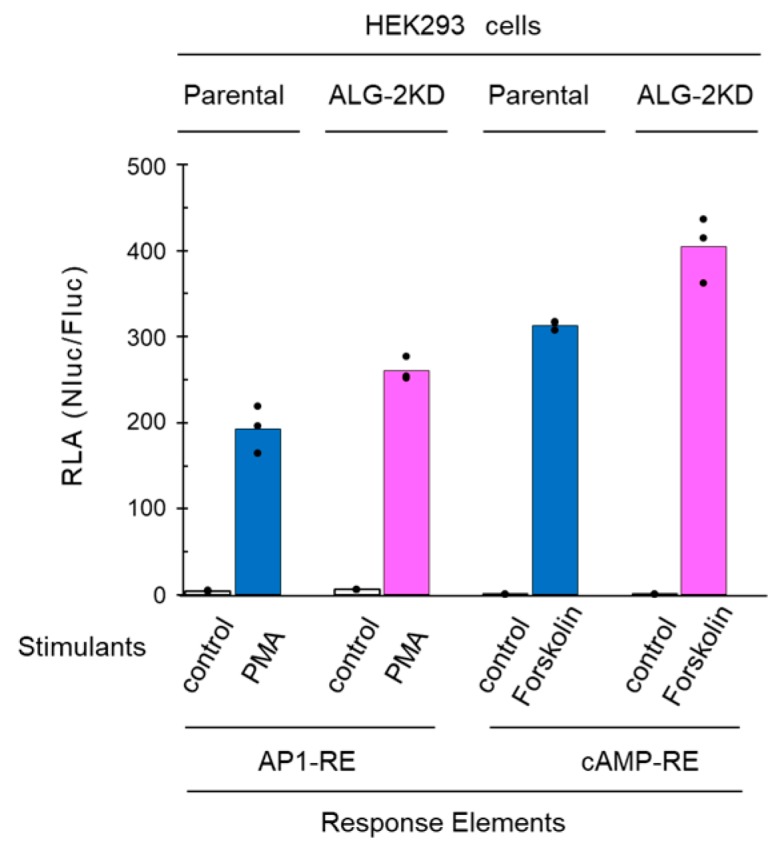
Comparison of reporter activities of AP-1 response element (AP1-RE) and cyclic AMP response element (cAMP-RE) between the parental and ALG-2KD HEK293 cells. The Ca^2+^-independent NanoLuc reporters directed by AP1-RE and by cAMP-RE were analyzed in the parental and ALG-2KD HEK293 cells by stimulation with PMA (10 ng/mL; vehicle, 0.01% DMSO) and forskolin (10 μM; vehicle, 0.1% DMSO) for 6 h in triplicate assays. Dots and bars represent individual and averaged values of RLA obtained from triplicate assays, respectively.

In order to exclude the possibility that ALG-2 deficiency influences the post-transcriptional processing of luciferase mRNAs or stability of translated luciferases, Ca^2+^-independent NanoLuc reporter assays were performed using an AP1-RE (AP-1 response element) construct and a cAMP-RE (cAMP response element) construct (Figure 5). The AP1-RE reporter gave a marked increase in RLA by stimulation with a PKC activator, PMA (10 ng/mL). Although the RLA values of the HEK293 ALG-2KD cells tend to be greater than those of the parental HEK293 cells, the difference was relatively small (average RLA: 260 vs. 193, 35% increase in the ALG-2KD cells). Similarly, upon stimulation with a cAMP-inducing reagent, forskolin (10 μM), the cAMP-RE-directed RLA values increased dramatically, but difference of the RLA values was small between the ALG-2KD cells and parental HEK293 cells (average RLA: 404 vs. 313, 29% increase in the ALG-2KD cells).

### 2.5. More Enhancement of the Reporter Activity in the ALG-2KD Cells by Carbachol

**Figure 6:**
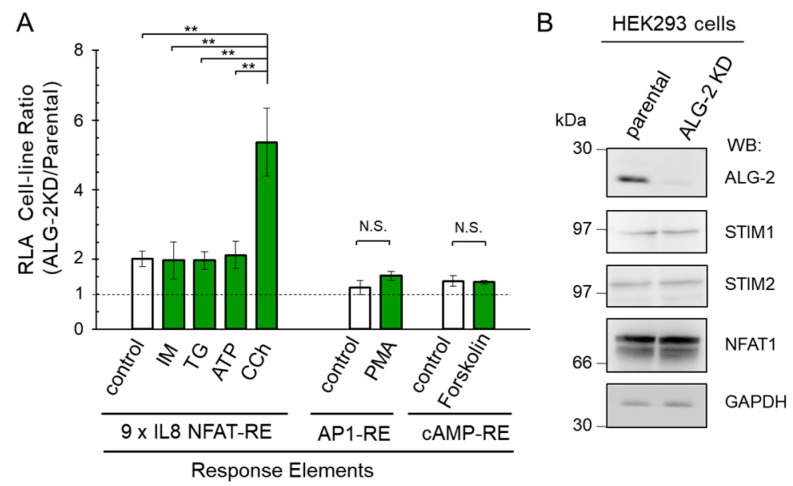
Preferentially enhanced NFAT-RE reporter activities in the ALG-2KD cells by stimulation with carbachol. (**A**) The RLA ratios between the ALG-2KD and the parental HEK293 cells obtained from more than three independent experiments were calculated and presented as mean ± SEM (*n* = 3 for IM, TG, and ATP; *n* = 4 for CCh; *n* = 7 for control using NFAT-RE; *n* = 3 for control, PMA and forskolin using AP1-RE or cAMP-RE). Statistical significance by Tukey’s test is indicated by asterisks (**, *p* < 0.01). N.S., not significant. IM, ionomycin (1 μM); TG, thapsigargin (200 nM); CCh, carbachol (100 μM). (**B**) Total cell lysates from the parental and ALG-2KD HEK293 cells were analyzed by Western blotting (WB) using indicated antibodies.

As shown in Figure 4, the HEK293 ALG-2KD cells gave greater RLA values than the parental HEK293 cells, but the degrees of differences were various depending on stimuli. To statistically evaluate the differences of RLA between the two cell-lines, we repeated the experiments more than three times, and ratios of average RLA values obtained from each triplicate assay were calculated and summarized in Figure 6A. The cell-line RLA ratios were approximately 2 for basal activity (control, resting condition), stimulation with ionomycin (IM), thapsigargin (TG), and ATP. On the other hand, the ALG-2KD cells gave a greater cell-line RLA ratio for carbachol (CCh, ratio 5.4 ± 1.0). In contrast, the cell-line RLA ratios were less than 1.5 in both AP1-RE- and cAMP-RE-directed assays (PMA and forskolin, respectively), and the differences were not statistically significant (N.S.) between the unstimulated state and the stimulated state. These results indicate that the ALG-2KD cells exhibit higher Ca^2+^-regulated transactivation activity of NFAT, specifically, downstream of carbachol-induced Ca^2+^ signaling than the parental HEK293 cells. Since carbachol links to SOCE for Ca^2+^ mobilization, we investigated whether there are differences in the amounts of SOCE-associated proteins and NFATs between the parental HEK293 cells and the ALG-2KD cells. Western blotting with respective antibodies gave similar intensities for STIM1, STIM2 and NFAT1 (Figure 6B). Clear specific bands could not be observed for Orai1 and SARAF in both cell lines under the Western blot conditions used.

### 2.6. Enhanced Ca^2+^-Mobilization by Carbachol in the ALG-2KD Cells

**Figure 7:**
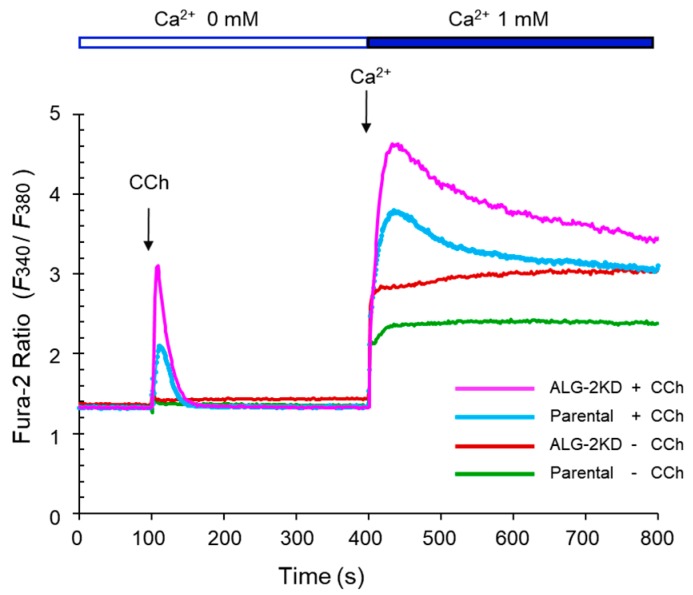
Augmented Ca^2+^ mobilization in the ALG-2KD cells detected by Fura-2 fluorescence ratiometry. The parental HEK293 and ALG-2KD cells were loaded with Fura-2 AM, and ratiometric analysis using a spectrofluorometer was performed as described in Materials and Methods. Ratio of 510 nm-fluorescence intensity (*F*_340_/*F*_380_) by alternate excitation at 340 and 380 nm was recorded. Cells suspended in a Ca^2+^-free Ringer’s solution containing 0.01 mM EGTA were stimulated with carbachol (+CCh, 100 μM) or mock treated (−CCh) at 100 s, followed by addition of Ca^2+^ to the cell suspension (final concentration, 1 mM) at 400 s to initiate Ca^2+^ entry from the extracellular space.

To investigate whether the enhanced 9×IL8 NFAT-RE reporter activities in the ALG-2KD cells compared to the parental HEK293 cells correlate with differences in Ca^2+^-mobilization capacities between the two cell lines, we performed Fura-2 ratiometric analysis with a spectrofluorometer equipped with a temperature controlled cuvette holder. As shown in Figure 7, addition of carbachol solution (final concentration, 100 μM) to the constantly stirred Fura-2AM-loaded cell suspensions in a Ca^2+^-free Ringer’s solution at 37 °C induced a transient increase in the ratio of 510 nm-fluorescence intensities (*F*_340_/*F*_380_) by alternate excitation at 340 and 380 nm. The *F*_340_/*F*_380_ value was higher in ALG-2KD cells than in the parental HEK293 cells. Addition of Ca^2+^ (final 1 mM) to the cell suspension induced elevation of *F*_340_/*F*_380_ in all conditions, but carbachol-stimulated cells gave higher values, suggesting acceleration of SOCE by depletion of the ER-stored Ca^2+^ by the carbachol-GPCR-IP_3_-IP_3_R pathway. The ALG-2KD cells showed higher *F*_340_/*F*_380_ values than the parental HEK293 cells under the carbachol-stimulated condition. These results of the Ca^2+^ measurement using a Fura-2 Ca^2+^ indicator are consistent with the enhanced Nluc responses upon carbachol stimulation in the ALG-2KD cells using the 9×IL8 NFAT-RE reporter assay (Figure 4). The ALG-2KD cells may have a larger capacity for GPCR-IP_3_ responses and SOCE.

### 2.7. Enhancement of Carbachol-Stimulated NanoLuc Response by Exogenous Expression of NFATs

HA-tagged human NFATs (HA-NFAT1-4) were transiently expressed by co-transfection of each expression plasmid with the reporter vectors. As shown in Figure S6A,B, although NFAT1 showed the highest RLA values by carbachol stimulation, the RLA value without stimulation was relatively high and resulted in the smaller calculated fold stimulation in both HEK293 cells and ALG-2KD cells compared to the greater fold stimulation by NFAT3 and NFAT4. NFAT2 isoform A (isoA) has a C-terminal truncation compared to isoform C (isoC) [2]. Both NFAT2 isoforms showed higher RLA values without carbachol stimulation under the condition used. The higher reporter activities in the presence and absence of carbachol by exogenously expressed HA-NFATs partly correlate with expression levels of NFATs as suggested by WB analyses of transfected cells with an anti-HA antibody (WB signal intensity: NFAT1, NFAT2 isoA, isoC > NFAT3 >> NFAT4) (Figure S6C). It remains unknown, however, whether the unexpected extremely weak WB signal for HA-NFAT4 was due to a low mRNA stability, a low translation efficiency, a rapid degradation of the entire protein or removal of an N-terminal region including the HA-tag by unidentified proteases.

**Figure 8:**
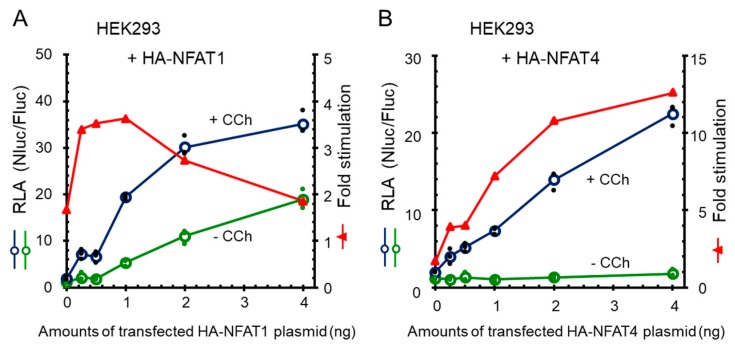
Greater carbachol-dependent reporter activity by NFAT4 than by NFAT1. HEK293 cells were transfected with various amounts of HA-tagged NFAT expression plasmids (0–4 ng) for (**A**) NFAT1 or (**B**) NFAT4. Empty vector was supplemented to equalize the total amounts of pCMV-3×HA-A vector and its derivatives (4 ng). Cells were stimulated with carbachol (+CCh, 100 μM) or unstimulated (−CCh) for 6 h before luciferase assays. Small dots and unfilled circles represent individual and averaged RLA values obtained from triplicate assays, respectively. Ratio of RLA values obtained in the presence and absence of carbachol [RLA(+CCh)/RLA(−CCh) ] was calculated and expressed as fold stimulation (red line graphs).

To investigate whether the carbachol-dependent and -independent 9×IL8 NFAT-RE reporter gene expressions correlate with expressed amounts of NFAT, we transfected HEK293 cells with increasing amounts of expression plasmids for HA-NFAT1 or HA-NFAT4. Expression of HA-NFAT1 caused increase in both carbachol-dependent and -independent reporter activities, and fold stimulation decreased beyond 1 ng of transfected plasmid (Figure 8A). On the other hand, the carbachol-independent activity was maintained at a low level by HA-NFAT4, resulting in a greater fold stimulation than HA-NFAT1 (Figure 8B). Decrease in fold stimulation by HA-NFAT4 was not observed up to 4 ng of the transfected plasmid, indicating that NFAT4 is a better transcription factor for the newly developed reporter gene system. To determine the minimum time required to detect the Nanoluc reporter gene activation by NFAT, HEK293 cells were co-transfected with the reporter plasmids and HA-NFAT4 expression plasmid, and stimulated with carbachol (Figure S7). An unequivocal increase in the RLA values was observed at as early as 2 h after stimulation. The RLA values reached maximum at 6 h, and then slightly decreased at 8 h.

### 2.8. Dose Dependency of the Carbachol Stimulated Reporter Gene Expression

**Figure 9:**
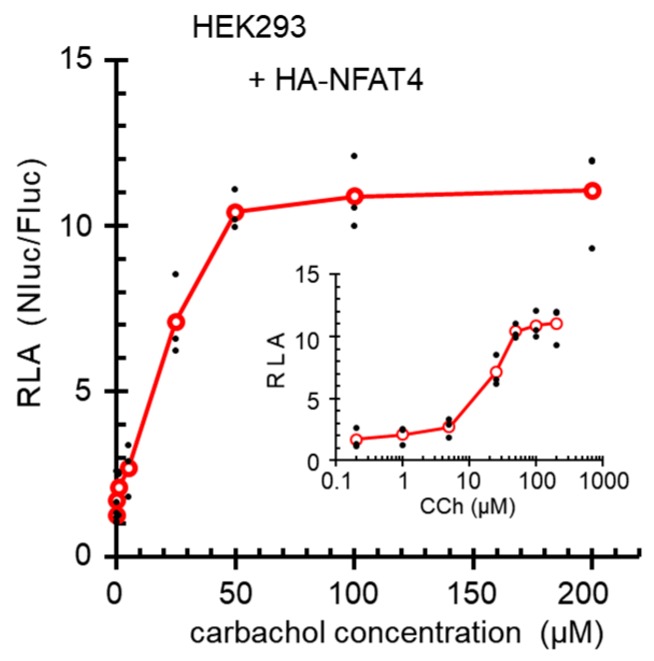
Dose-dependency of the carbachol-stimulated reporter gene activities. HEK293 cells seeded in 24-well plates were co-transfected with pCMV-3×HA-A/NFAT4 (1 ng), pGL4.53[*luc2/*PGK] (20 ng), and pNL3.2[*NlucP*/9×IL8 NFAT-RE/minP] (50 ng). One day after transfection, cells were unstimulated or stimulated with different concentrations of CCh (0.2–200 μM) for 6 h. Small dots and unfilled circles represent individual and averaged RLA values obtained from triplicate assays, respectively. A graph in semi-logarithmic scale of CCh concentrations is presented in inset.

To investigate dose-dependency of the Nanoluc reporter gene activation by carbachol stimulation, HEK293 cells were co-transfected with the reporter plasmids and HA-NFAT4 expression plasmid, and stimulated with different concentrations of carbachol (Figure 9). Carbachol stimulated the reporter gene expression in a dose-dependent fashion up to 50 μM, and the estimated half maximal effective concentration (EC_50_) was approximately 20 μM.

## 3. Discussion

Translocation of NFAT from the cytoplasm to the nucleus is regulated by calcineurin, a Ca^2+^/CaM-dependent Ser/Thr-phosphatase [1,7], which dephosphorylates multiple phosphorylated sites in the N-terminal regulatory region of NFAT, resulting in exposure of the nuclear localization signal [2,8]. Nuclear translocation of NFAT by fluorescent microscopic analysis of GFP-fused NFAT or immunostaining of endogenous NFAT has been used as a marker to demonstrate cytosolic Ca^2+^ elevation upon cell stimulation as biological endpoint effects [8,50,51]. Reporter gene assays are convenient to monitor biological effects of chemical compounds in a cell-based system. However, requirement of partner transcription factors (Jun and Fos as AP1) that are activated through different signaling pathways has limited the usage of the well-documented non-palindromic *IL2* NFAT-RE reporter gene assays when researchers desire to focus on Ca^2+^ responses. In this study, we have developed a new Ca^2+^-dependent reporter gene system by designing NanoLuc expression directed by nine-tandem repeats of a pseudo-palindromic *IL8* NFAT-RE (Figure 1 and Figure S1). HEK293 cells, used in the present study, were established from a human embryonic kidney [52] and have been shown to express neuronal markers including a muscarinic type 3 (M_3_) AChR [46,53] and to respond to an AChR agonist carbachol [54,55]. We have demonstrated that the newly developed NanoLuc reporter can be used to monitor Ca^2+^ signaling upon stimulation with commonly used Ca^2+^-mobilizing reagents including ionomycin (IM, Figure 2) and thapsigargin (TG, Figure 3) as well as carbachol (Figure 4, Figure 8 and Figure 9, Figures S6 and S7).

As summarized in Figure 6A, compared to the parental HEK293 cells, the ALG-2KD cells showed higher responses to stimulants, particularly carbachol. While no significant differences were observed between the parental HEK293 cells and the ALG-2KD cells in the AP1-RE-directed or cAMP-RE-directed NanoLuc reporter assays by stimulation with PMA and forskolin, respectively, ALG-2 deficiency enhanced the expression of the Ca^2+^-dependent NanoLuc reporter gene preferentially from the NFAT-RE by carbachol stimulation. This fact suggests selective involvement of ALG-2 in Ca^2+^ signaling or homeostasis. Carbachol binds to M_3_ AChR and activates the G-protein coupled receptor pathway, leading to the generation of inositol 1,4,5-trisphosphate (IP_3_) and induction of Ca^2+^-release from the ER. Because carbachol/ATP-stimulated enhancement of RLA was suppressed by BTP2 (a CRAC channel inhibitor) (Figure 4), the observed enhancement of NFAT-RE responses in the ALG-2KD cells appeared to depend on a pathway involved in the induction of SOCE [44,45], in which STIM1 and Orai1 play key roles [24,25,26]. Analysis of Ca^2+^ mobilization by carbachol using a Fura-2 ratiometric Ca^2+^ indicator suggests augmented Ca^2+^-release from the ER and enhancement of SOCE in the ALG-2KD cells (Figure 7). It is not known how Ca^2+^-independent NFAT-RE reporter activity is also enhanced in the ALG-2KD cells compared to the parental HEK293 cells (Figure 6A, unstimulated control condition). A lack of Ca^2+^-buffering capacity by ALG-2 is a potential explanation. However, Fura-2 ratiometric analysis suggests similar Ca^2+^ concentrations in the cytosol under unstimulated conditions between the two cell lines (Figure 7). Although ALG-2 requires Ca^2+^ to interact with target proteins and interaction is disrupted in the presence of EGTA (a calcium chelator), addition of Ca^2+^ into cell lysates is not necessary to detect interaction in vitro binding assays [35,36]. ALG-2 might associate with known or unknown proteins to suppress the NFAT-RE-dependent gene expression in the presence of a trace amount of Ca^2+^ in the unstimulated condition.

CHERP (named after “calcium homeostasis and endoplasmic reticulum protein”) was first reported as an ER-transmembrane protein regulating Ca^2+^ homeostasis in human erythroleukemia (HEL) and Jurkat T cells [56,57]. However, we and others demonstrated that a majority of the CHERP protein resides in the nucleus by associating with spliceosome factors [36,58]. Although CHERP interacts with ALG-2 and regulates alternative splicing of the IP_3_R1 pre-mRNA [36], the effect of the ALG-2 knockdown alone on the alternative splicing was very small (less than 3% in the isoform production) [36]. Thus, contribution of ALG-2 to Ca^2+^ homeostasis modulation observed in the present study is unlikely to depend on CHERP. We speculate that ALG-2 modulates Ca^2+^-homeostasis dependent on the interaction with other known or unknown cytoplasmic or membrane proteins. Interestingly, ALG-2 interacts with mucolipin-1 (MCOLN1, also known as TRPML1), a lysosomal membrane resident ion channel [37]. Release of Ca^2+^ from the lysosome triggers calcineurin-dependent nuclear translocation of transcription factor EB (TFEB), which regulates autophagy and lysosome biogenesis [59]. However, the observed enhancement of NFAT-RE responses in the ALG-2KD cells may not depend on the lysosomal MCOLN1-Ca^2+^-calcineurin pathway, since BTP2 (a CRAC channel inhibitor) suppressed the enhanced RLA by carbachol/ATP in the ALG-2KD cells (Figure 4A).

X-ray crystallographic studies have revealed that ALG-2 recognizes two different ALG-2-binding motifs (ABM-1 and ABM-2) fitting to different hydrophobic pockets [48,60,61]. Interestingly, a negative SOCE regulator SARAF [47] has an ABM-2 sequence in the cytosolic domain and the recombinant glutathione-*S*-transferase (GST) protein fused with the eight-residue peptide from the SARAF sequence binds to ALG-2 in in vitro binding assays [48]. We attempted to determine interaction between the endogenous proteins of ALG-2 and SARAF, but we failed to detect endogenous SARAF in HEK293 cells by Western blotting using commercially available antibodies. Future studies including preparation of highly specific antibodies against SARAF are necessary to elucidate the mechanism of the plausible ALG-2-dependent Ca^2+^ homeostasis modulation in HEK293 cells. Intriguingly, the ALG-2KD cells exhibited a higher basal NanoLuc reporter activity than the parental HEK293 cells under the resting condition (Figure 6A, control). We transiently expressed FLAG-ALG-2 in the ALG-2KD cells and measured NanoLuc activities after stimulation with carbachol (Figure S8), but we have not been able to confidently obtain results that show suppressive effects of overexpressed FLAG-ALG-2 compared to FLAG-ALG-2 E47A/114A, a mutant defective in both Ca^2+^-binding and interaction with binding partners [62,63]. Since ALG-2 interacts with a variety of proteins that should influence the NFAT-RE reporter assays in positive and negative fashions with different degrees, improvement of the reporter assay conditions by expressing appropriate amounts of FLAG-tagged or untagged ALG-2 at appropriate times before stimulation would be necessary to evaluate the biological effects of ALG-2 on the Ca^2+^ signaling and Ca^2+^ homeostasis. Since the HEK293 ALG-2KD cells were established about ten years ago [49], we could not exclude the possibility that genetic and/or epigenetic changes in some Ca^2+^-homeostasis-related genes might have arisen during passages of this cell line. Alternatively, unexpected off-target effects might have been elicited by the shRNA expression. Results of Western blot analysis indicate no significant changes in the expressed amounts of STIM1, STIM2, and NFAT1 proteins between the parental HEK293 cells and ALG-2KD cells (Figure 6B), but other SOCE- or NFAT-regulatory proteins such as calcineurin have remained unexamined. The RLA values obtained in the present study were small in the parental HEK293 cells with endogenous NFAT proteins, but they were markedly enhanced by co-transfection with NFAT expression plasmids (Figure 2B, Figure 8 and Figure S6). Greater values of fold stimulation (RLA with stimulation vs. RLA without stimulation) by NFAT4 than other NFATs may be explained as follows: the amount of exogenously expressed HA-NFAT4 was very low (Figure S6), and it may be sufficiently phosphorylated after de novo synthesis to be kept localized in the cytoplasm until calcineurin-catalyzed dephosphorylation. On the other hand, expression levels of other HA-NFATs may be in excess and over capacities of regulatory kinases, and unphosphorylated NFATs may translocate into the nucleus without Ca^2+^ signaling as suggested by a decrease in fold stimulation when larger amounts (2 and 4 ng) of HA-NFAT1 expression plasmid were used for transfection (Figure 8). Faster export of NFAT4 from the nucleus to the cytoplasm by re-phosphorylation in the nucleus [50,51] and requirement of additional nuclear Ca^2+^ mobilization for NFAT4 activation [51] may also account for the lower carbachol-independent reporter activity. Moreover, basal NFAT activities may be influenced by unintended Ca^2+^ mobilization of the HEK293 cells during cell culture manipulation such as trypsin treatment [45] and medium changes that accompany mechanical stress [64].

In the present study, we developed a reporter gene assay based on Ca^2+^-dependent transcription factor activation. In conclusion, the reporter gene activities were dependent on the time after stimulation with carbachol (Figure S7) and on the carbachol concentrations (Figure 9), and were influenced by the amounts of exogenously expressed NFATs (Figure 8). Although the molecular mechanisms of the enhancing effects of carbachol in the ALG-2KD cells on the reporter gene expression still need to be clarified, the newly developed NanoLuc NFAT-RE reporter system should provide a useful tool for basic research in Ca^2+^ signaling and for searching and evaluation of cell stimulating chemical compounds.

## 4. Materials and Methods

### 4.1. Antibodies and Reagents

Rabbit polyclonal antibody against ALG-2 was prepared as described previously [65]. The following antibodies were used for Western blotting: anti-STIM1 (ab57834) from Abcam (Cambridge, UK); anti-STIM2 from Alomone labs; anti-Orai1 (G-2, sc-377281), anti-GAPDH (6C5, sc-32233) from Santa Cruz Biotechnology (Dallas, TX, USA); anti-NFAT1 (D43B1, #5861S) from CST Japan (Tokyo, Japan); HRP-conjugated goat antibodies against mouse IgG and rabbit IgG from Jackson Immunoresearch (West Gove, PA, USA). Reagents of biochemical or cell culture grade were purchased as follows: ionomycin from Cayman Chemical (Ann Arbor, MI, USA); BTP2 from Calbiochem/Merck Millipore (Temecula, CA, USA); carbachol (sc-202092) from Santa Cruz Biotechnology; forskolin, FK506, phorbol 12-myristate 13-acetate (PMA), and thapsigargin from Wako (Osaka, Japan); puromycin and blasticidin from InvivoGen (San Diego, CA, USA). A calcium assay Fura-2 kit was purchased from Dojindo (Kumamoto, Japan).

### 4.2. Plasmid Construction

The NanoLuc reporter plasmid of NFAT-RE containing nine tandem repeats of the pseudo-palindromic NFAT binding site located upstream of the *IL8* promoter (5′-GGAATTTCC-3′, [19,22]), designated pNL3.2[*NlucP*/9×IL8 NFAT-RE/minP], was constructed by a stepwise insertion of a synthetic DNA fragment containing three NFAT-RE repeats into the upstream multiple cloning site of pNL3.2[*NlucP*/minP] vector (#N1041, Promega) as follows: (i) insertion of a pair of oligonucleotides (5′-ctgagctcgctagcttgaggaatttccattgaggaatttccattgaggaatttccatta-3′ and 5′-gatctaatggaaattcctcaatggaaattcctcaatggaaattcctcaagctagcgagctcaggtac-3′; NFAT-RE underlined) between the KpnI and BglII sites; (ii) insertion of a pair of oligonucleotides (5′-gatcgttgaggaatttccattgaggaatttccattgaggaatttccattagatctcgagtgga-3′ and 5′-agcttccactcgagatctaatggaaattcctcaatggaaattcctcaatggaaattcctcaac-3′; a new BglII site double-underlined) between the BglII and HindIII sites, where the upstream BglII site was destructed and a new BglII site was created; (iii) repetition of the 2nd step.

The NanoLuc reporter plasmid of AP1-RE containing six units of the AP1-binding site (5′-TGAG/CTCA-3′; see Ref. [12]), designated pNL3.2[*NlucP*/AP1-RE/minP], was constructed by inserting a synthetic DNA fragment corresponding to 272-338 nt of pGL4.44[*luc2P*/AP1/Hygro] (Promega; GenBank acc. No. JQ858516) between the KpnI and XhoI sites of pNL3.2[*NlucP*/minP] using a pair of oligonucleotides (5′-ctgagctctgagtcagtgactcagtgagtcagtgactcagtgagtcagtgactcagc-3′ and 5′-tcgagctgagtcactgactcactgagtcactgactcactgagtcactgactcagagctcaggtac-3′; 5′-TGAGTCA-3′ and 5′-TGACTCA-3′, underlined and double-underlined, respectively). The NanoLuc reporter plasmid containing the cyclic AMP response element (cAMP-RE, also known as CRE), designated pNL[*NlucP*/CRE/Hygro], was constructed at Promega upon our request and we purchased it. To eliminate potential adverse effects of the SV40 early enhancer/promoter used for expression of the hygromycin resistance gene on the cAMP-RE-dependent transcription activation, a KpnI/EcoRI fragment containing the cAMP-RE sequence and a part of the NlucP cDNA sequence was transferred to the KpnI/EcoRI sites of pNL3.2 [*NlucP*/minP]. The resultant plasmid was designated pNL3.2 [*NlucP*/CRE/minP].

Mouse NFAT1 cDNA plasmids of pENTR11 WT NFAT1 and pENTR11 CA NFAT1 (constitutively active: multiple serine to alanine mutations in the SRR and SPXX repeat motifs in the NFAT1 regulatory domain) were gifts from Dr. Anjana Rao (Addgene plasmid #11791 and #11792). The 3-kb HindIII/XbaI fragment containing the NFAT1 ORF was each subcloned into the HindIII/XbaI sites of pcDNA3. See Supplementary Material for the construction of human HA-tagged NFAT expression vectors.

### 4.3. Cell Culture and Retrovirus Infection

HEK293 cells were cultured in DMEM (Nissui, Tokyo, Japan) supplemented with 4 mM glutamine, 5% fetal bovine serum (FBS), 100 units/mL penicillin and 100 μg/mL streptomycin at 37 °C under humidified air containing 5% CO_2_. HEK293 ALG-2KD cells that were established by constitutive expression of the short hairpin RNA specific for ALG-2 [49] were maintained with the medium containing 1 μg/mL of puromycin. Human cDNAs of STIM1 and Orai1 (GenBank IDs, BC021300 and BC015369, respectively) were purchased from Open BioSystems/GE Dharmacon (Lafayette, CO, USA) and subcloned into pCX4bsr and pCX4pur [66], respectively. HEK293 cells stably co-expressing STIM1 and Orai1 (HEK293 S1/O1) were generated by retroviral infection essentially as described previously [32]. Retroviruses were prepared from culture media of PLAT-A cells [67] (kindly provided by Dr. Toshio Kitamura, The University of Tokyo) that had been transfected with pCX4pur-Orai1 and pCXbsr-STIM1 using FuGENE 6 (Invitrogen) according to the manufacturer’s instructions. Cells expressing respective genes were selected with antibiotics (1 μg/mL puromycin, 5 μg/mL blasticidin), and antibiotics-resistant cells were pooled and maintained in the medium containing respective antibiotics.

### 4.4. Reporter Assay

HEK293 cells, HEK293 ALG-2KD cells or HEK293 S1/O1 cells were seeded one day before transfection in 24-well plates (2.5 × 10^4^ cells/well) in 0.5 mL of the medium without antibiotics used for stable cell selection (puromycin or blasticidin) and were co-transfected with the pNL3.2(*NlucP*/minP)-derived reporter vector (50 ng) and pGL4.53 [luc2/PGK] (firefly luciferase gene driven by the phosphoglycerate kinase gene promoter; Promega #E5011) (20 ng) using FuGENE 6 and cultured for 24 h. When effects of exogenous NFAT expressions were investigated, pcDNA3-based NFAT-expression plasmids (1 ng) were also used for co-transfection. Then, cells were stimulated with respective reagents for 6 h, followed by lysis with passive lysis buffer (PLB). Enzymatic reactions of Fluc and Nluc were carried out using a kit for Nano-Glo^®^ Dual-Luciferase Reporter Assay System (Promega) according to the manufacturer’s instructions. Luminescence was measured with AB-2250 luminescencer MCA (ATTO, Tokyo, Japan) at room temperature without filter to collect all emitted lights (ranging wavelengths: 350–670 nm) to acquire data presented in Figure 2, Figure 3, Figure 4, Figure 5 and Figure 6, Figures S4 and S6–S8). Alternatively, a multimode microplate reader Mithras^2^ LB943 (Berthold Technologies, Bad Wildbad, Germany) was used at 25 °C with wide ranging wavelengths (380–650 nm) to acquire data presented in Figure 8 and Figure 9, Figures S2 and S3. NanoLuc (Nluc) activity was normalized against firefly luciferase activity (Fluc) reflecting transfection efficiency and expressed as relative luciferase activity (RLA), the ratio of Nluc to Fluc (Nluc/Fluc).

### 4.5. Fura-2 Assay

Measurements of cytosolic free Ca^2+^ were performed by a Fura-2 fluorescence ratiometric assay using a Spectrofluorophotometer RF-5300PC (Shimadzu, Kyoto, Japan) equipped with a temperature controlled cuvette holder. One day before the Fura-2 assay, HEK293 cells or HEK293 ALG-2KD cells were seeded in 12-well plates (3.5 × 10^5^ cells/well) in 1 mL of the medium without antibiotics that had been used for stable cell selection and maintenance (1 μg/mL puromycin). Cells were loaded with Fura-2 by incubation with a Ringer’s solution (5 mM HEPES-NaOH pH 7.4, 155 mM NaCl, 4.5 mM KCl, 2 mM CaCl_2_, 1 mM MgCl_2_, 10 mM glucose) containing 4 μM Fura-2 AM and 0.04% Pluronic F-127 for 30 min at 37 °C in the dark. Then, the cells were rinsed with the Ca^2+^-free Ringer’s solution containing 0.1 mM EGTA twice, and were resuspended with the Ca^2+^-free Ringer’s solution containing 0.01 mM EGTA. A suspension of the Fura-2 loaded cells was stirred in a black quartz cuvette and maintained at 37 °C. Fluorescence emission at 510 nm by alternate excitation at 340/380 nm was operated and recorded by Super Ion Probe Software version 1.00 (Shimadzu, Kyoto, Japan). Sequential addition of reagents during recording was carried out by a syringe injection through a septum.

### 4.6. Western Blotting

Proteins were resolved by SDS-PAGE and transferred to polyvinylidene difluoride (PVDF) membranes by the semi-dry blotting method. Then, membranes were blocked for 1 h at room temperature with 5% skimmed milk in PBST [PBS (137 mM NaCl, 2.7 mM KCl, 8 mM Na_2_HPO_4_ and 1.5 mM KH_2_PO_4_, pH 7.4) containing 0.1% Tween 20], followed by incubation with primary antibodies in PBST containing 0.1% bovine serum albumin (BSA) at 4 °C overnight. After washing three times (5 min each time) with PBST, membranes were incubated with secondary antibodies in PBST containing 0.1% BSA at room temperature for 1 h with gentle agitation, then washed three times with PBST and once with PBS. For WB analysis of Orai1, blocking was performed with PBST containing 1% skim milk, the primary antibody was diluted with PBST containing 5% BSA, and the secondary antibody was diluted with PBST containing 1% skimmed milk. For WB analysis of NFAT1 and NFAT4, TBST (25 mM Tris-HCl, pH 7.4, 150 mM NaCl, 0.1% Tween-20) was used instead of PBST: membranes were blocked with 5% skimmed milk, probed with the primary antibodies diluted with TBST containing 5% BSA, and the secondary antibody diluted with TBST containing 5% skimmed milk. The primary antibody dilution rates used in this study were as follows: Orai1 1:100; GAPDH, 1:2,000; ALG-2, 1:3,000; STIM1, STIM2, and NFAT1. Signals were detected by the chemiluminescence method using either Super Signal West Pico Chemiluminescent Substrate (Thermo Fisher Scientific, Rockford, IL, USA) or Immobilon Western Chemiluminescent HRP Substrate WBKLS0500 (Merck Millipore, Temecula, CA, USA) and analyzed with LAS-3000mini (Fuji Film, Tokyo, Japan).

### 4.7. Statistical Analysis

Statistical analysis was performed by one-way analysis of variance (ANOVA) followed by Tukey’s test for agonist or inhibitor data sets using Origin 9.1 (Microcal Software, Northampton, MA, USA). *P*-values less than 0.05 are considered statistically significant.

## Supplementary Materials

Supplementary materials can be found at http://www.mdpi.com/1422-0067/19/2/605/s1.

## Abbreviations

AChR	acetylcholine receptor
ALG-2KD	ALG-2 knockdown
cAMP-RE	cyclic AMP response element
CRAC	Ca^2+^-release activated Ca^2+^
IP_3_R	inositol trisphosphate receptor
NanoLuc	nanoluciferase
Nluc	NanoLuc
RLA	relative luciferase activity
Fluc	firefly luciferase
PMA	phorbol 12-myristate 13-acetate
RE	response element
SOCE	store-operated calcium entry
