# Supplementary material for: Nanoluciferase Reporter Gene System Directed by Tandemly Repeated Pseudo-Palindromic NFAT-Response Elements Facilitates Analysis of Biological Endpoint Effects of Cellular Ca2+ Mobilization
Source: Int J Mol Sci. 2018 Feb 18;19(2):605. doi: 10.3390/ijms19020605 (PMC5855827; doi:10.3390/ijms19020605)
Supplement: Supplementary file 1 [file ijms-19-00605-s001.zip › Suppl_Figures.pdf]

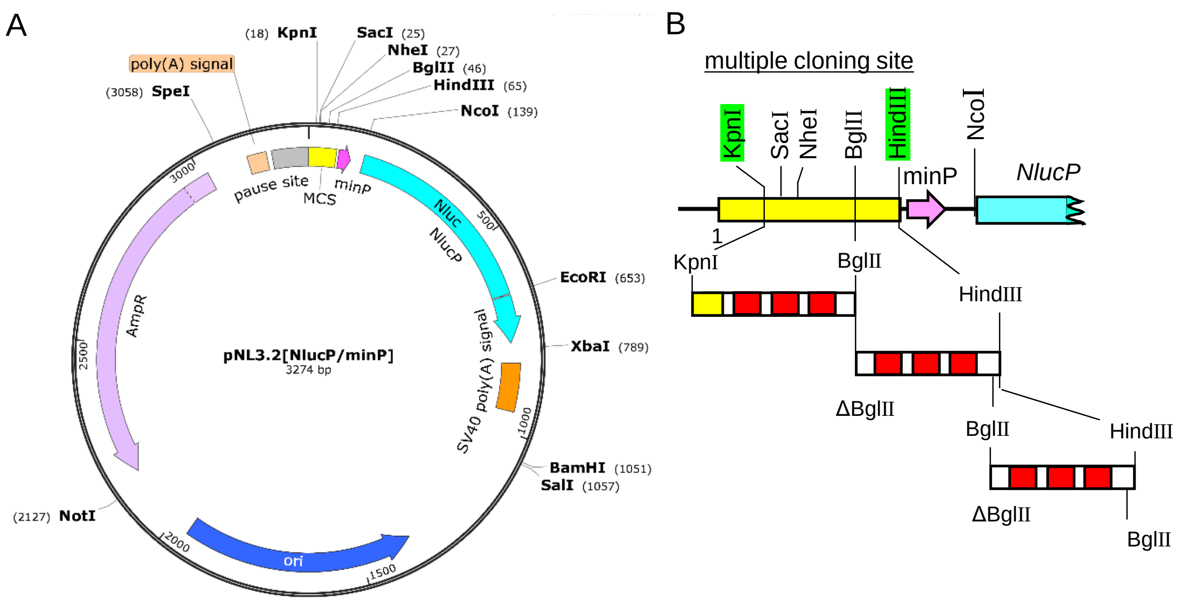

C pNL3.2[*NlucP*/9xIL8 NFAT-RE/minP]

```
1- GGCCTAACTGGCCGGTACCtgagctcgctagcttgaggaatttccattgaggaatttccattga
   ggaatttccattagatcgttgaggaatttccattgaggaatttccattgaggaatttccattag
   atcgttgaggaatttccattgaggaatttccattgaggaatttccattagatctcgagtggaAG
   CTTAGACACTAGAGGGTATATAATGGAAGCTCGACTTCCAGCTTGGCAATCCGGTACTGTTGGT
   AAAGCCACCATGGTCTTCACACTCGAAGATTTCGTTGGGGACTGGCGACAGACAGCCGGCTACA-320
            M  V  F  T  L  E  D  F  V  G  D  W  R  Q  T  A  G  Y
            └──► NlucP
```

Supplementary Figure S1. Construction of pNL3.2[*NlucP*/9xIL8 NFAT-RE/minP]. (A) Map of pNL3.2[*NlucP*/minP] (GenBank Acc. No. JQ513370.1). The vector has a minimal TATA-box promoter (minP) with low basal activity for measuring the activity of transcriptional response elements (REs) using destabilized NanoLuc luciferase (NlucP) containing a PEST sequence at the C-terminus. The plasmid map was drawn with SnapGene® Viewer 4.1.4. A yellow box indicates multiple cloning sites (MCS). (B) Stepwise insertion of tandemly repeated NFAT-REs. Synthetic oligonucleotides containing 9-tandem repeats of the *IL8* NFAT-RE (ggaatttccc, indicated by red boxes) were inserted between the KpnI (GGTAC/C) and HindIII (A/AGCTT) sites located in the MCS of pNL3.2[*NlucP*/minP] by three steps. ΔBglII indicates the BglII site (A/GATCT) destructed by the oligonucleotide insertion. (C) A partial nucleotide sequence of pNL3.2[*NlucP*/9xIL8 NFAT-RE/minP]. Inserted oligonucleotides are indicated by lower case letters, where the *IL8* NFAT-RE (ggaatttcc) is indicated by red letters. Boldface, repeated sequences; thin black underline, destructed BglII site; thick green underline, BglII site. Highlighted in yellow, sequence from the MCS; green, KpnI and HindIII sites; pink, minP; cyan, partial *NlucP* sequence. The N-terminal amino acid sequence of NlucP is presented under the nucleotide sequence. A supplementary material file containing the plasmid map of pNL3.2[*NlucP*/9xIL8 NFAT-RE/minP], similar to the map presented in panel A, can be viewed by a free software SnapGene® Viewer, which is available from the following URL: http://www.snapgene.com/products/snapgene_viewer/.

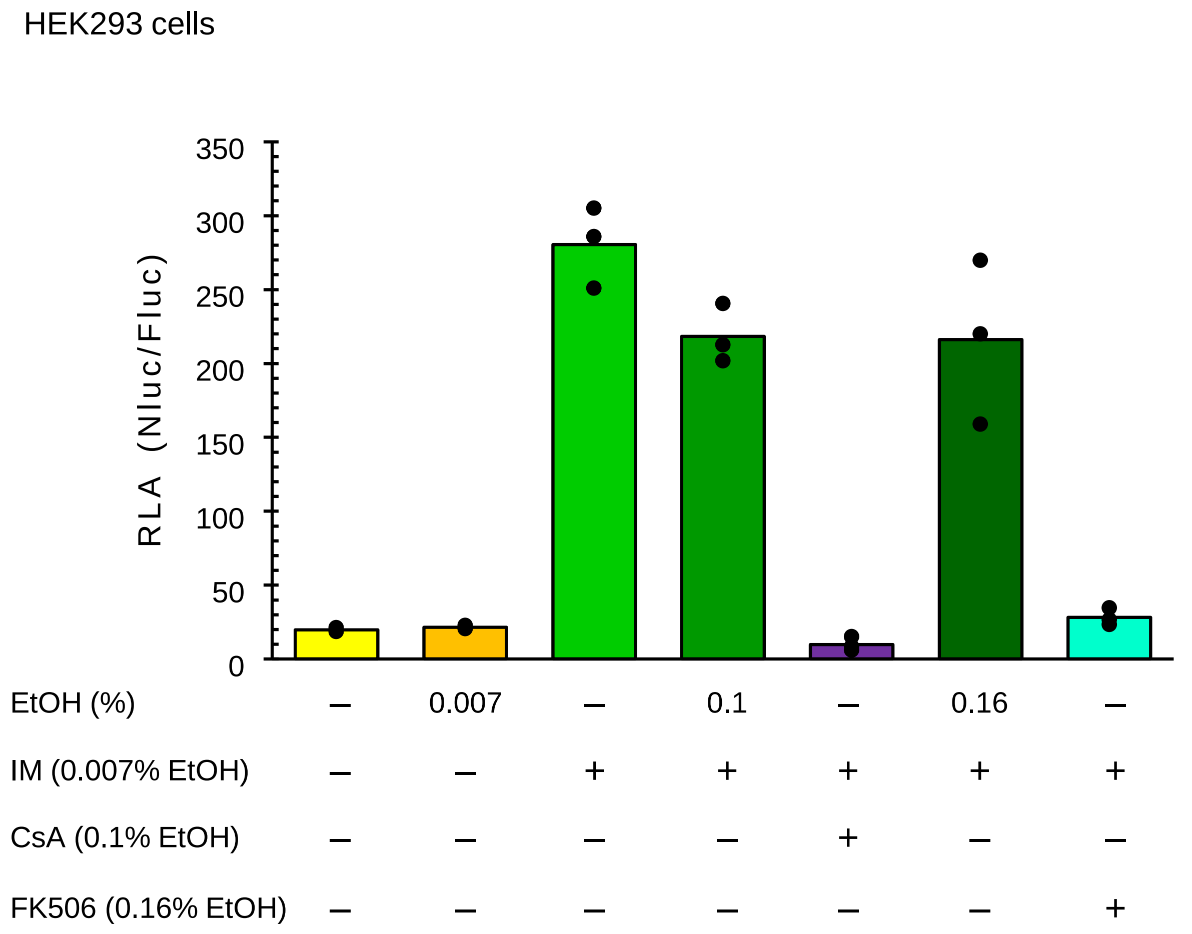

Supplementary Figure S2. Inhibitory effects of calcineurin inhibitors on the 9xIL8 NFAT-RE reporter activities. HEK293 cells were co-transfected with 1 ng of a wild type murine NFAT1 expression plasmid (pcDNA3-mNFAT1 WT), pGL4.53[*luc2*/PGK] (20 ng), and pNL3.2[*NlucP*/9xIL8 NFAT-RE/minP] (50 ng). Cells were treated with vehicles (different concentrations of ethanol, EtOH), ionomycin, cyclosporine A (CsA), and FK506. Final concentrations of ethanol in the culture media were 0.007% (ionomycin), 0.1% (CsA), and 0.16% (FK506). Dots and bars represent individual and the averaged values obtained from triplicate assays, respectively.

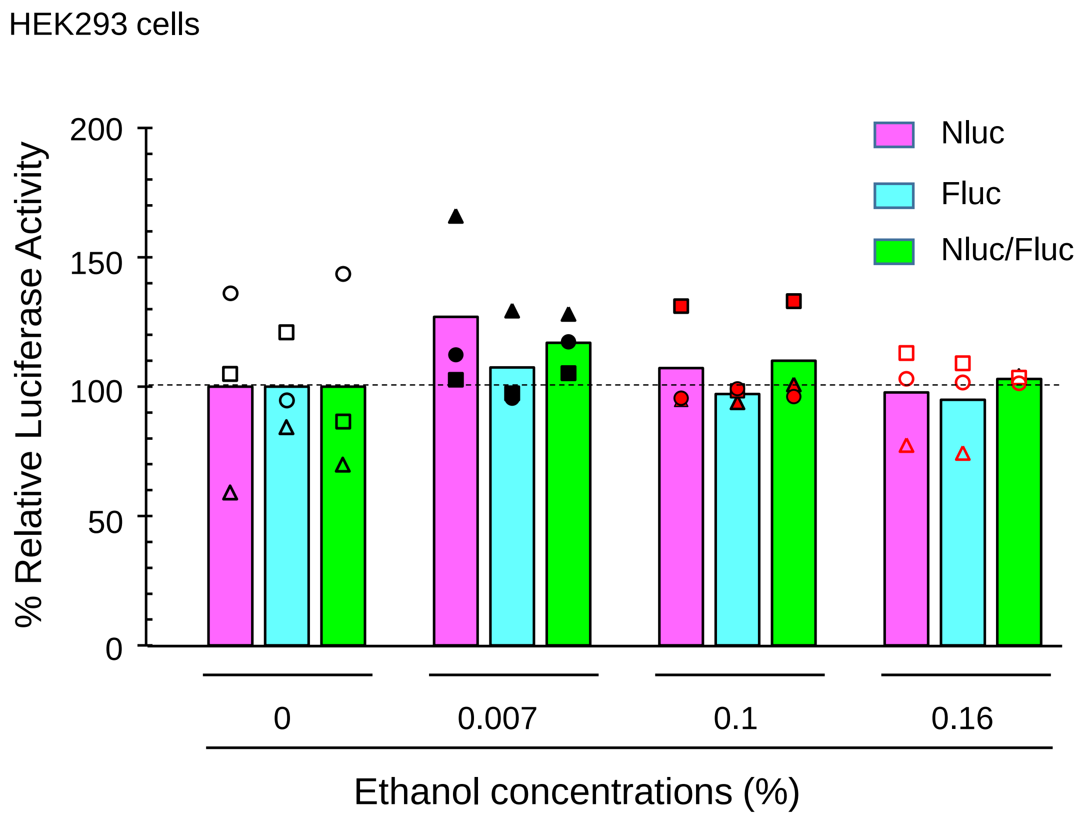

Supplementary Figure S3. No adverse effects on the 9xIL8 NFAT-RE reporter activities by treatment of cells with low concentrations of ethanol. HEK293 cells were co-transfected with pcDNA3-mNFAT1 WT (1 ng), pGL4.53[*luc2*/PGK] (20 ng), and pNL3.2[*NlucP*/9xIL8 NFAT-RE/minP] (50 ng). Reporter assays were performed in triplicates for each condition by treatment with different concentrations of ethanol that was used as vehicles for dissolving ionomycin, cyclosporine A (CsA), and FK506. Final concentrations of ethanol in the culture media were 0.007% (ionomycin), 0.1% (CsA), and 0.16% (FK506). After 6 h, cells were harvested and luciferase activities were measured. In the presented bar graph, average values of Nluc activity, Fluc activity and Nluc/Fluc ratio obtained under the condition of no ethanol treatment are expressed as 100% of relative luciferase activity. Individual values obtained from the triplicate assays in each condition are represented by three different symbols, where the same symbols indicate the identical cell samples used for measuring luciferase activities and calculation of Nluc/Fluc ratios. Differences of % relative luciferase activities were small between no ethanol treatment and ethanol treatment, suggesting that cell toxicity by ethanol alone is negligible under the conditions used.

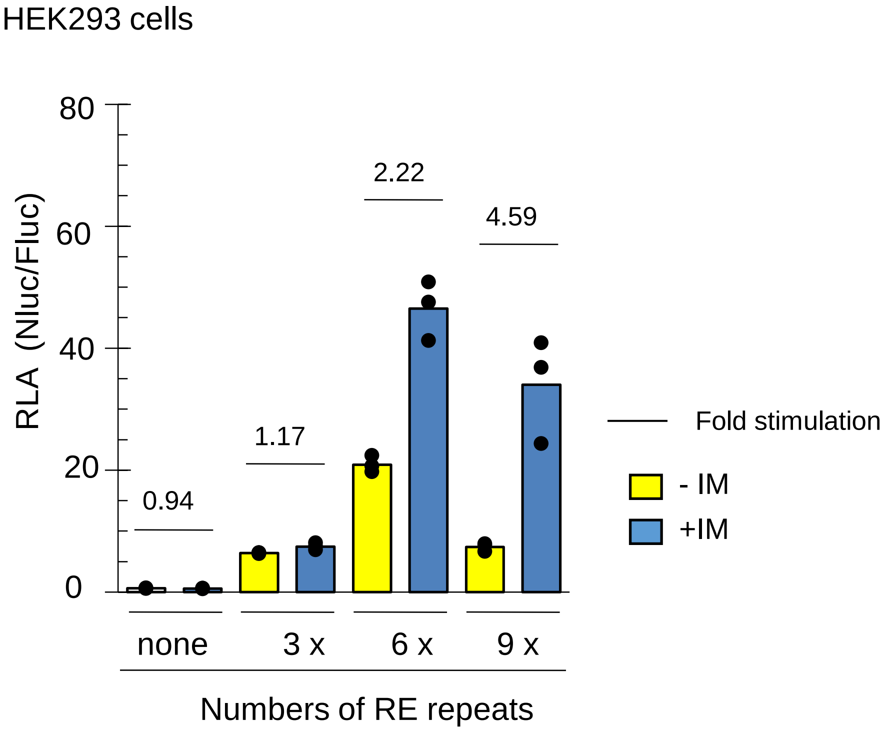

Supplementary Figure S4. Evaluation of the NanoLuc reporter system by ionomycin treatment in the absence of exogenously expressed NFAT. Reporter assay was performed using endogenous NFAT, i.e., similar to Figure 2 except for transfection with NFAT expression plasmid. HEK293 cells were transfected with a firefly luciferase (Fluc) expression plasmid (pGL4.53[*luc2*/PGK]) (50 ng) and pNL3.2[*NlucP*/minP] (20 ng) containing different numbers (none, 3, 6 and 9) of the *IL8* NFAT-RE. One day after transfection, cells were stimulated with ionomycin (IM, 1 μM; vehicle, 0.007% ethanol) for 6 h. Cell lysates were used to measure luminescent signals of NanoLuc (Nluc) and Fluc using a Nano-Glo Dual-Luciferase Reporter Assay System. The ratio of Nluc to Fluc, Nluc/Fluc, is expressed as normalized relative luciferase activity (RLA). Dots and bars represent individual and the averaged RLA values obtained from triplicate assays, respectively.

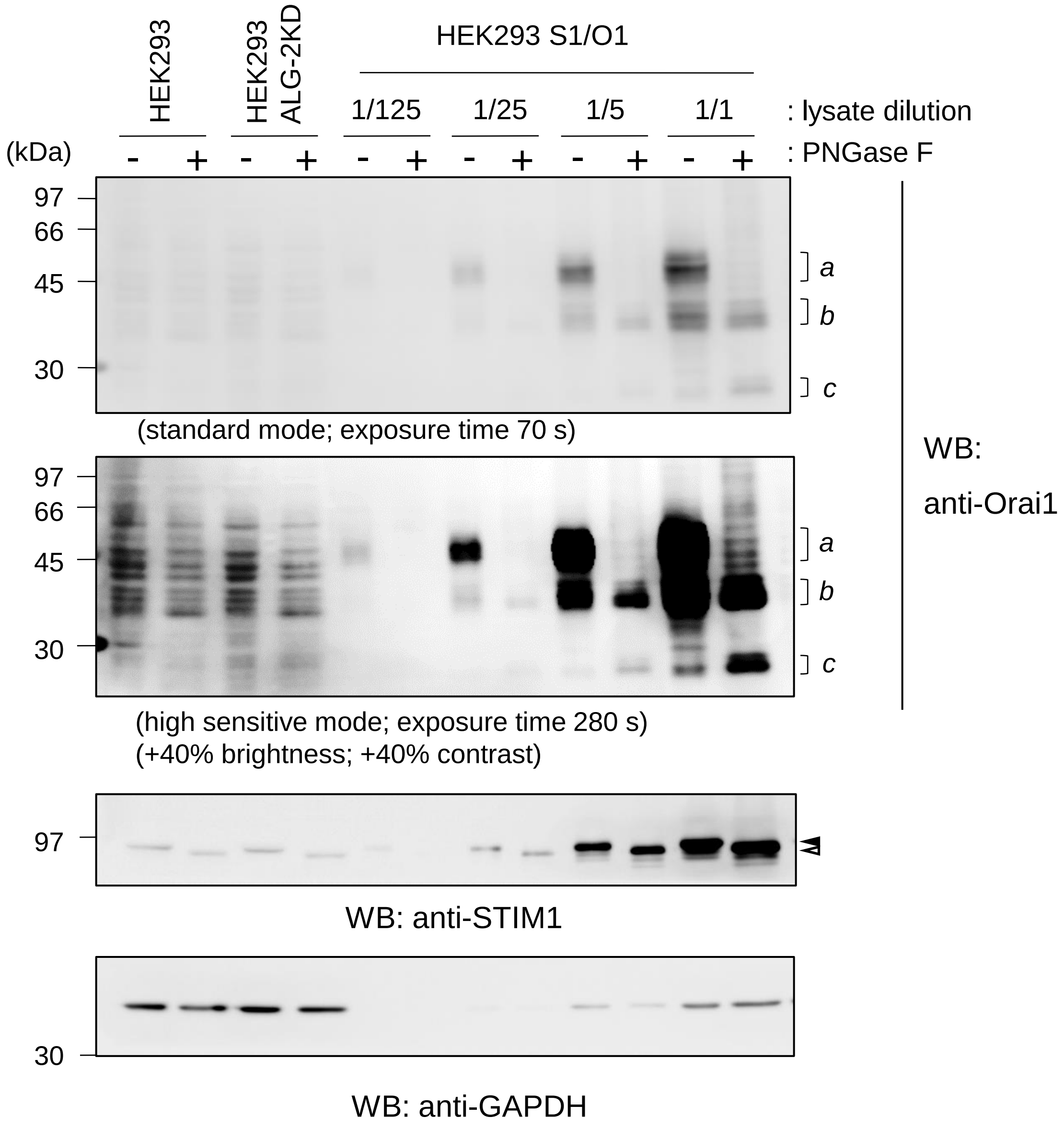

Supplementary Figure S5. Electrophoretic mobility shift of overexpressed Orai1 by deglycosylation with PNGase F. Cell lysates of the parental HEK293, ALG-2KD and S1/O1 cells were treated or untreated with PNGase F as described in the Supplementary Materials and Methods. The SDS-PAGE sample prepared from the S1/O1 cell lysates were serially diluted (x5, x25 and x125) with SDS-PAGE sample buffer. Western blotting (WB) was performed with antibodies against Orai1, STIM1, and GAPDH (a loading control) as indicated, respectively, and analyzed with LAS-3000mini. Chemiluminescent images for anti-Orai1 were obtained under two different conditions: *top panel*, standard mode with exposure time of 70 s; *2nd panel*, high sensitive mode with exposure time of 280 s, followed by image processing with increases in brightness (+40%) and contrast (+40%). Multiple bands specifically immunoreactive with anti-Orai1 are indicated by half-square brackets *a*, *b*, and *c*. The PNGase F treatment also caused a small shift of STIM1 band, as indicated from a closed to open arrow head.

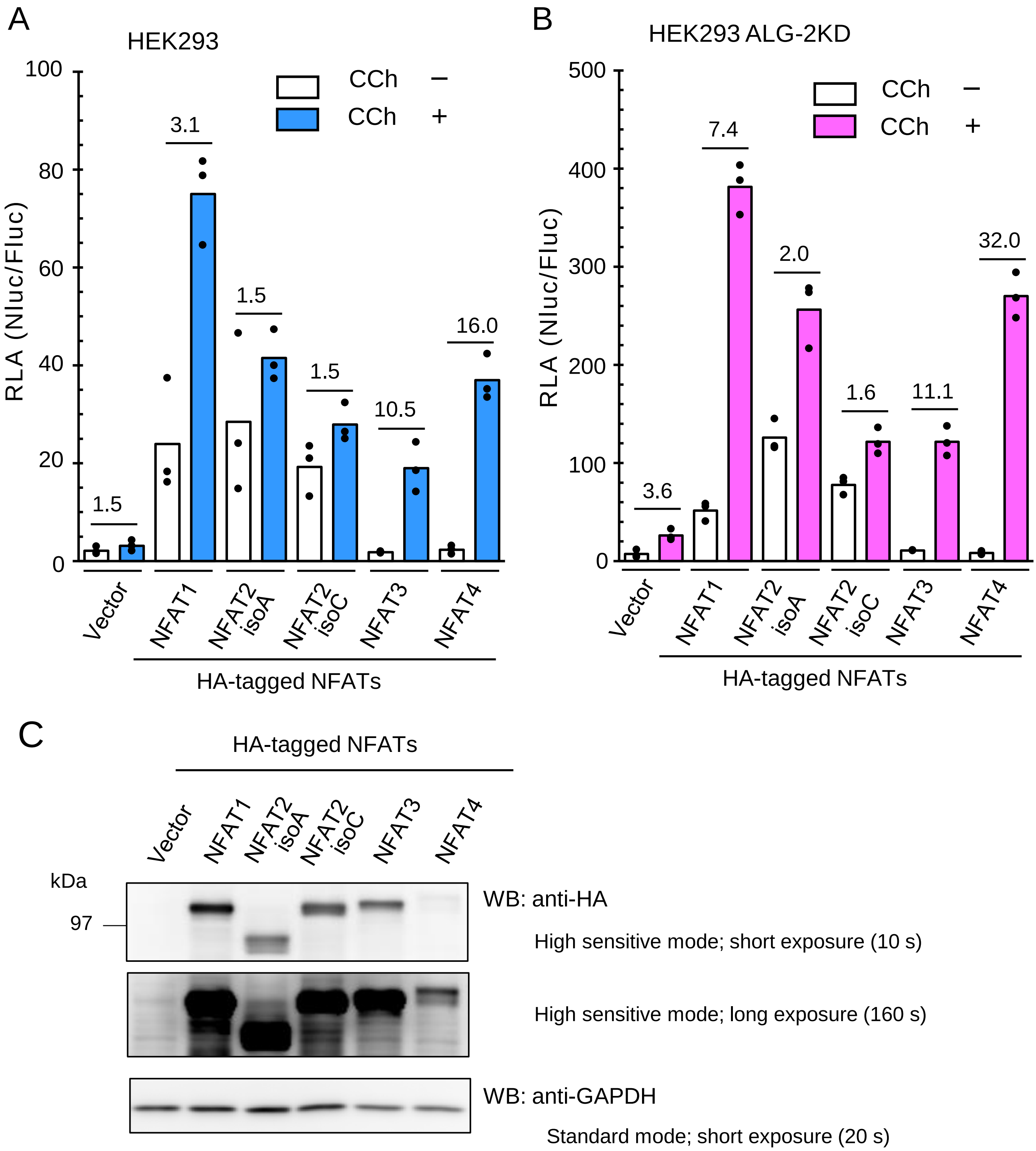

Supplementary Figure S6. Different effects of exogenously expressed NFAT isoforms on the 9xIL8 NFAT-RE reporter. HEK293 (A) and ALG-2KD (B) cells were co-transfected with the plasmids of 9xIL8 NFAT-RE NanoLuc reporter (50 ng) and Fluc (20 ng), and expression vector (1 ng) for HA-tagged NFAT1, NFAT2 isoform A (isoA), NFAT2 isoform C (isoC), NFAT3 or NFAT4. Cells were treated with or without carbachol (CCh, 100 μM) for 6 h, and the reporter assay was performed. Dots and bars represent individual and the averaged values of RLA in triplicate assays, respectively. Fold stimulation was calculated and the averaged values were indicated above the bars. (C) HEK293 cells seeded in a 24-well plate were transfected with expression vectors for indicated HA-tagged NFATs (50 ng plasmid). Total cell lysates were analyzed by Western blot (WB) with indicated antibodies. Chemiluminescent signals were detected under indicated image acquisition conditions with LAS-3000mini (Fuji Film). Expressions of HA-tagged NFATs were not detected by WB when smaller amounts of the plasmids (1 ng) were used for transfection.

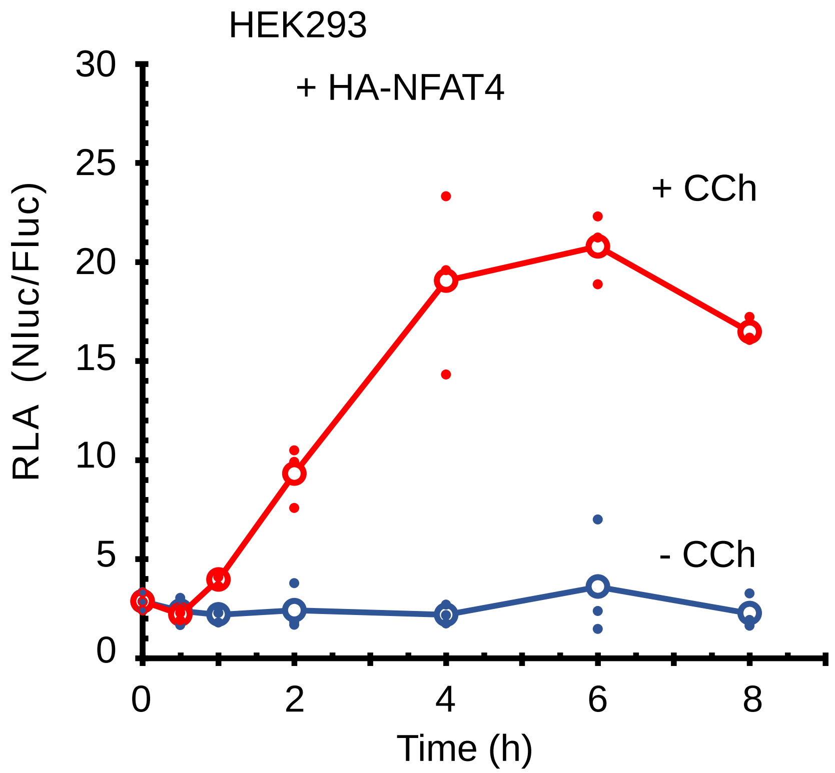

Supplementary Figure S7. Time course of the carbachol-stimulated reporter gene activities. HEK293 cells seeded in 24-well plates were co-transfected with pCMV-3xHA-A/NFAT4 (1 ng), pGL4.53[*luc2*/PGK] (20 ng), and pNL3.2[*NlucP*/9xIL8 NFAT-RE/minP] (50 ng). One day after transfection, cells were stimulated with or without carbachol (CCh, 100 μM; vehicle, medium), and culture was continued for a different period of time (0, 0.5, 1, 2, 4, 6, and 8 h) in triplicates. Cells were harvested at indicated time and subjected to luciferase activities. Dots and bars represent individual and the averaged values of RLA in triplicate assays, respectively. A line graph is presented for CCh treatment (+CCh) and no CCh treatment (-CCh).

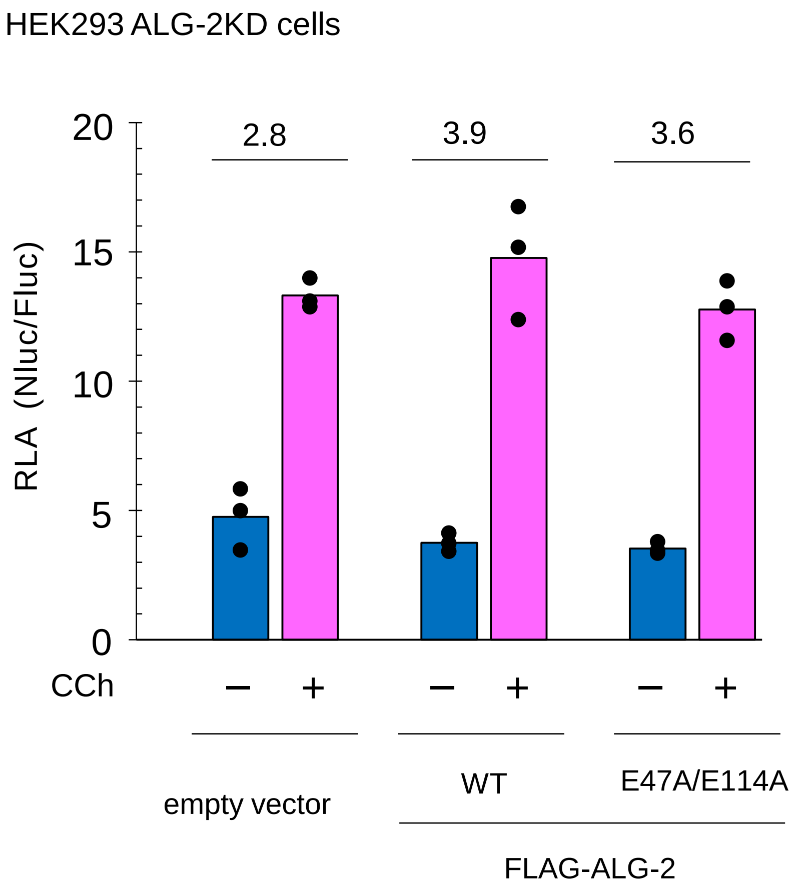

Supplementary Figure S8. No suppressive effects of overexpression of ALG-2 on carbachol-dependent 9xIL8 NFAT-RE reporter activity in the ALG-2 knockdown (ALG-2KD) HEK293 cells. Cells were co-transfected with 100 ng of expression vectors for FLAG-tagged ALG-2 wild type (WT), Ca^{2+}-binding site defective mutant (E47A/E114A), or empty vector as a control together with 25 ng of pNL3.2[*NlucP*/9xIL8 NFAT-RE/minP] and 20 ng ng pGL4.53 [luc2/PGK]. One day after transfection, cells were treated with or without carbachol (100 μM) for 6 h and subjected to luciferase assays. Dots and bars represent individual and the averaged values of RLA in triplicate assays, respectively. Fold stimulation was calculated and the averaged values were indicated above the bars.
