# Supplementary material for: Nanoluciferase Reporter Gene System Directed by Tandemly Repeated Pseudo-Palindromic NFAT-Response Elements Facilitates Analysis of Biological Endpoint Effects of Cellular Ca2+ Mobilization
Source: Int J Mol Sci. 2018 Feb 18;19(2):605. doi: 10.3390/ijms19020605 (PMC5855827; doi:10.3390/ijms19020605)
Supplement: Supplementary file 1 [file ijms-19-00605-s001.zip › Suppl_Material & Methods.pdf]

Supplementary Materials and Methods

Plasmid construction

pCMV-3xHA-A

The HA-tag expression vector pCMV-3xHA-A was constructed by first digesting pCMV-3xFLAG-A (see Ref. 1) with NotI and BamHI to remove a 3xFLAG-encoding DNA fragment and inserting a pair of 2xHA-encoding oligonucleotides (5′-ggccgccaccatggtttacccatacgatgttcctgactatgctggctatccctatgacgtcccggactatgcag-3′; 5′-gatcctgcatagtccgggacgtcatagggatagccagcatagtcaggaacatcgtatgggtaaaccatggtggc-3′) and then by inserting a pair of 1xHA-encoding oligonucleotides (5′-gatcttatccatatgacgttccagattacgctccgg-3′; 5′-gatcccggagcgtaatctggaacgtcatatggataa-3′) at the BamHI/BglII site. The manipulation was done by a previous laboratory member (Dr. Yuki Maemoto).

HA-tagged human NFAT expression vectors

Human NFAT cDNAs were obtained from different sources. NFAT1 cDNA clone (GenBank ID, BC136418) was purchased from Open BioSystems/GE Dharmacon (Lafayette, CO, USA). NFAT2 cDNA clones FLJ76687 (encoding 1-716 a.a. of UniProt ID O95644-2, NFATc1 Isoform A-alpha; GenBank ID, AK292641), FLJ61216 (encoding 473-943 a.a. of UniProt ID O95644-1, NFATc1 Isoform C-alpha; GenBank ID, AK293760) and NFAT4 cDNA clone FLJ77451 (GenBank ID, AK290370) were obtained from NITE Biological Resource Center (NBRC) (Kisarazu, Japan). pEGFP-C1 NFAT3 was a gift from Dr. Toren Finkel (Addgene plasmid #10961) [2]. To construct a full length NFAT2 isoform C-alpha encoding 1-943 a.a., the overlap PCR method was used to fuse 1-503 a.a. with 495-943 a.a. using two pairs of primers (template: FLJ76687; Pair 1, forward, 5′-ttacgctccgggatccccaagcaccagctttccagt-3′, reverse, 5′-<u>tggacacggtcttccctgtgatgcggtg</u>-3′; template: FLJ77451; Pair 2, forward, 5′-<u>caccgcatcacagggaagaccgtgtcca</u>-3′, reverse, 5′-cggtatcgataagcttctaggagtgggtgctcgtgc-3′; overlapped primers encoding 495-503 a.a. underlined). The cDNA fragments encoding each NFAT were amplified by PCR and subcloned into the BamHI/HindIII site of pCMV-3xHA-A with an In-Fusion® HD Cloning Kit (Clontech/Takarabio) using each pair of specific primers (NFAT1, 5′-ttacgctccgggatccaacgcccccgagcggca-3′, 5′-cggtatcgataagcttttacgtctgatttctggcag-3′; NFAT2 isoform C-alpha, 5′-ttacgctccgggatccccaagcaccagctttccagt-3′, 5′-cggtatcgataagcttctaggagtgggtgctcgtgc-3′; NFAT3, 5′-ttacgctccgggatccggggcggccagctgcgagga-3′, 5′- cggtatcgataagctttcaggcaggaggctcttctc-3′;

NFAT4, 5′-ttacgctccgggatccactactgcaaactgtggcgc-3′, 5′-cggtatcgataagcttttagagcccatcagatcttc-3′). pCMV-3xHA-A/NFAT2 isoform A-alpha was constructed by transferring a BamHI/SalI fragment of pEF-BOS-EX/NFAT2 (1-716) containing the NFAT2 cDNA that had been subcloned by the In-Fusion PCR using pEGFPC1-huNFATc1EE-WT (a kind gift from Dr. Jerry Crabtree, Addgene plasmid #24219) [3] as a template with a pair of specific primers (5′- ggccgcccggggatccatgtcaagcaccagctttc-3′; 5′-tcactctagagtcgactcagaaaaagcaccccacg-3′) for expression of untagged NFAT2 isoform A-alpha using the EF1α promoter vector pEF-BOS-EX (kindly provided from Dr. Shigekazu Nagata, deposited at RIKEN BRC, ID RDB07940) [4].

PNGase F Treatment

HEK293 cells, HEK293 ALG-2 KD cells, and HEK293 S1/O1 cells grown in 100-mm dishes were washed once with ice-cold PBS and lysed by sonication with 200 μL of lysis buffer (20 mM HEPES-NaOH pH 7.4, 142.5 mM KCl, 1.5 mM $MgCl_2$, 0.2% NP-40) containing 1 mM PMSF, 3 μg/ml leupeptin, 1 μM pepstatin A, 0.1 mM Pefabloc, and 10 μM E-64. Protein concentrations of total cell extracts were determined using a Pierce™ BCA Protein Assay Kit (Thermo Scientific #23225) according to the manufacturer's instructions. An aliquot of total cell extracts (9 μL, 15.7 μg proteins) was processed for deglycosylation with 1 μL of N-Glycosidase F (PNGase F, New England BioLabs #P0704S) according to the manufacturer's instructions except that the incubation reaction was performed overnight instead of 1 h.

References

1. Katoh, K.; Shibata, H.; Suzuki, H.; Nara, A.; Ishidoh, K.; Kominami, E.; Yoshimori, T.; Maki, M. The ALG-2-interacting protein Alix associates with CHMP4b, a human homologue of yeast Snf7 that is involved in multivesicular body sorting. *J. Biol. Chem.* **2003**, *278*, 39104-39113.
2. Ichida, M.; Finkel, T. Ras regulates NFAT3 activity in cardiac myocytes. *J. Biol. Chem.* **2001**, *276*, 3524-3530.
3. Beals, C.R.; Clipstone, N.A.; Ho, S.N.; Crabtree, G.R. Nuclear localization of NF-ATc by a calcineurin-dependent, cyclosporin-sensitive intramolecular interaction. *Genes Dev.* **1997**, *11*, 824-834.
4. Mizushima, S.; Nagata, S. pEF-BOS, a powerful mammalian expression vector. *Nucleic Acids Res.* **1990**, *18*, 5322.
